# A critical review of “Internet addiction” criteria with suggestions for the future

**DOI:** 10.1556/JBA.3.2014.4.1

**Published:** 2014-12-18

**Authors:** ANTONIUS J. VAN ROOIJ, NICOLE PRAUSE

**Affiliations:** ^1^IVO Addiction Research Institute; ^2^Erasmus MC, The Netherlands; ^3^Department of Psychiatry, University of California, Los Angeles

**Keywords:** Internet use disorder, Internet addiction, diagnostic criteria, DSM-5, behavioral addiction, addictive disorders

## Abstract

*Aims:* In the last 5 years a deluge of articles on the topic of Internet addiction (IA) has proposed many candidate symptoms as evidence of this proposed disease. We critically reviewed the current approach to the measurement and identification of this new excessive behavior syndrome. *Methods:* Three popular models of IA were discussed: Griffith’s components model; Young’s Internet Addiction Test (IAT); and the criteria by Tao et al. (2010). We selected these models because they are widely cited and propose specific criteria for IA disorder. Our approach is not meant to provide an exhaustive review, but to discuss and critique the most salient trends in the field. *Results:* The models of Internet addiction share some criteria, including feeling a loss of control over Internet use; ensuing psychological, social, or professional conflict or problems; and preoccupation when not using the Internet. Other criteria inconsistently mentioned include: mood management, tolerance, withdrawal, and craving/anticipation. The models studied here share the assumption that the Internet can produce a qualitative shift to a diseased state in humans. *Conclusions:* We critically discussed the above criteria and concluded that the evidence base is currently not strong enough to provide support for an Internet addiction disorder. Future research areas are suggested: (1) Focusing on common impaired dimensions, (2) exploring neuroimaging as a model building tool, and (3) identifying shifts in the rewarding aspects of Internet use. Given the lack of consensus on the subject of Internet addiction, a focus on problem behaviors appears warranted.

## INTRODUCTION

In recent years, the term “addiction” has been expanded beyond substance dependence to include non-substance-related behaviors that cause problems and impairment. Proposed “process” or “behavioral” addictions have included such varied themes as shopping; exercise; gaming; and forms of Internet-enabled behavior such as online video gaming, socializing through social media, and various forms of sexual behavior (Grant, Potenza, Weinstein & Gorelick, 2010; Griffiths, 2005, 2012; Kuss & Griffiths, 2011; Sussman, Lisha & Griffiths, 2011; van Rooij, 2011). Considerable research to support these new addictions appears to follow a “me too” approach as investigators test for similarities with substance addictions and impulse control disorders that already appear in diagnostic manuals (Heyman, 2009). Moreover, there exists a general lack of agreement regarding how excessive behavior syndromes are defined and described (Mudry et al., 2011).

Addiction to a substance and addiction to a behavior may look similar in their effects on behavioral patterns, emotions, and physiology. For example, people might engage in theft to buy heroin (Jarvis & Parker, 1989) or to finance problem gambling behaviors (Crofts, 2003). Numerous similarities between gambling disorder (GD) and substance use disorders (SUDs) have been demonstrated (e.g., Potenza, 2006), with these leading to the inclusion of GD alongside substance use problems in the fifth edition of the Diagnostic and Statistical Manual of Mental Disorders (American Psychiatric Association, 2013). GD is the first such behavioral addiction included under the DSM heading of “Substance Use and Addictive Disorders” (Petry & O’Brien, 2013). However, clear differences also exist between SUDs and behavioral addictions. Perhaps the main difference is that substances provide, by definition, physiological input beyond what the body can produce by behavior alone. Consequently, SUDs are marked by several physically oriented criteria such as tolerance and withdrawal; these criteria are not generally present in behavioral addictions. Thus, serious debate exists regarding the similarity of criteria for behavioral addictions and SUDs.

Consequently, SUDs typically include several physical criteria, such as tolerance and withdrawal. There is debate on their applicability to behavioral addictions. Where some authors argue that behavioral addictions should and do display withdrawal and tolerance (Demetrovics et al., 2012; Griffiths, 2005; Petry et al., 2014) their implementation is often quite different from that of the tolerance and withdrawal associated with SUDs. Tolerance and withdrawal are critiqued in the current manuscript (see below).

Within the field of behavioral addictions, the subject of Internet addiction (IA) is of considerable interest. A primary driver of this interest is the recent inclusion of the more specific “Internet gaming disorder” in the DSM-5 appendix in order to stimulate research (American Psychiatric Association, 2013; Block, 2008; LaRose, Lin & Eastin, 2003; Petry & O’Brien, 2013). In this work, we will critically review the current approach to the measurement and identification of the excessive behavior syndrome sometimes referred to as Internet addiction (IA). Ultimately, we argue that it is probably more useful to characterize individual differences that interact with environmental factors and lead to high Internet use, rather than diagnose Internet addiction.

## MODELS OF INTERNET ADDICTION

Three popular models of IA will be discussed: Griffiths components model (Griffiths, 2005); Young’s Internet Addiction Test (IAT) (Widyanto, Griffiths & Brunsden, 2011; Young, 1998a, 1998b); and the more recent diagnostic criteria by Tao et al. (2010). We selected these models for study because they propose specific criteria for IA disorder and are widely cited. As an example of their impact, Young’s IAT was recently used as the basis for a large (*N=* 11,956) European study of IA (Durkee et al., 2012). There are various other models and scales available, like the CIUS (Meerkerk, van den Eijnden, Vermulst & Garretsen, 2009), the OCS (Davis, Flett & Besser, 2002), and the (gaming oriented) POGQ (Demetrovics et al., 2012). Our approach is not meant to provide an exhaustive review of all currently available conceptual models of IA, but to discuss and critique the most salient trends. Thus, our approach resembles an empirically grounded critique of popular trends, rather than an exhaustive review.

The first model, by Griffiths, also known as the “components model” (Brown, 1993; Griffiths, 1996, 2005), posits that all addictions consist of six distinct and common components (i.e., salience, mood modification, tolerance, withdrawal, conflict, and relapse). Griffiths further argues that addictions are excessive behaviors that share key elements of biopsychosocial processes (Griffiths, 2005), with his component criteria originating from the gambling field described previously by Brown (1993). Numerous scales have been developed to assess Griffith’s criteria for many domains, such as work addiction and gaming addiction (Andreassen, Griffiths, Hetland & Pallesen, 2012; Lemmens, Valkenburg & Peter, 2009; Meerkerk et al., 2009; Terry, Szabo & Griffiths, 2004; van Rooij, Schoenmakers, van den Eijnden, Vermulst & van de Mheen, 2012). Using confirmatory factor analysis, the criteria were recently found to fit the large sample data collected with two popular Internet addiction scales (CIUS/AICA-S) quite well (Kuss, Shorter, van Rooij, Griffiths & Schoenmakers, 2013).

Secondly, the work by Young takes the established criteria for pathological gambling as a starting point and defines Internet addiction as a failure of personal impulse control that does not involve external substances (Young, 1998b). This failure is described by the following set of criteria: (1) a preoccupation with the Internet, (2) the need to use the Internet for increasing amounts of time, (3) unsuccessful efforts to stop using the Internet, (4) mood change when attempting to stop or cut down Internet usage, (5) staying online longer than intended, (6) jeopardizing of significant relationships or opportunities due to excessive Internet usage, (7) lying about Internet use, (8) using the Internet as an escape from problems or seeking to relieve bad mood states (Widyanto & McMurran, 2004). Young’s criteria note that only personal (non-work related) Internet use should be evaluated, and that addiction is thought to be present when a client reports experiencing five or more of the above eight criteria. Like Griffith’s model, these criteria and this cut-off score can be viewed as a direct translation of criteria for PG in the DSM-5 (American Psychiatric Association, 2013). Other than replacing gambling behaviors with Internet behaviors, the only notable difference is that the DSM mentions a 12-month period, while Young mentions no time-period.

Young’s conceptualization has been popularized through the expanded 20-item IAT proposed in the 1998 self-help book *Caught in the Net* (Young, 1998a). Widyanto and McMurran (2004) characterized the IAT psychomet-rically using factor analysis. Using a convenience sample (online recruitment, *N —* 86), they obtained six factors in their analysis for Young’s IAT scale: (1) salience, (2) excessive Internet use, (3) neglecting work, (4) anticipation, (5) lack of control, and (6) neglecting social life. While Widyanto and McMurran concede that a limitation of Young’s instrument is that its main source of validity is face validity, they ultimately conclude that the IAT is both reliable and worthwhile as a tool for assessing subjects’ level of Internet addiction.

Thirdly, Tao et al. (2010) developed their diagnostic criteria for IA by considering the clinical characteristics of a large group of Chinese patients thought to have IA, as reported by psychiatrist evaluators. Using this approach, and excluding patients with bipolar disorder and/or psychotic disorders, Tao et al. (2010) proposed the following set of criteria: (a) *symptom criteria* (both must be present): preoccupation and withdrawal symptoms; (b) *one or more of these criteria:* (1) tolerance, (2) persistent desire and/or unsuccessful efforts to control use, (3) continued use despite problems, (4) loss of other interests, (5) use of the Internet to escape or relieve dysphoric mood; (c) *clinically significant impairment criterion:* functional impairments (reduced social, academic, working ability), including loss of a significant relationship, job, educational or career opportunities. The criteria also include a *course criterion* (d): Duration of IA must have lasted for an excess of three months, with at least six hours of Internet usage (non-business/non-academic) per day.

The three sets of discussed criteria for IA (Griffiths, 2005; Tao et al., 2010; Young, 2003) contain some commonalities (see Table 1). All sets of criteria describe feelings of a lack of control over Internet use; ensuing psychological, social, or professional conflict or problems (including “excessive use"); and mental preoccupation or salience. Other relevant features are mentioned inconsistently across the three models: mood management, tolerance, withdrawal, and craving/anticipation.

Another commonality in the models is researchers’ tendency to address Internet activities as a singular entity. The Internet incorporates a variety of potential activities, as demonstrated by the high correlations between measures of IA and time spent on online activities such as online gaming and social network use (van Rooij, Schoenmakers, van den Eijnden & van de Mheen, 2010). In some ways, saying someone is addicted to the Internet is akin to arguing that somebody with a drinking problem is addicted to a liquor store. As such, IA is ambiguous in terminology or is even a misnomer (Starcevic, 2013). That said, most authors, including the authors of the models reviewed, continue the general use of IA as a descriptor of specific addictive behaviors associated with Internet use. This critique follows that same approach by reviewing Internet use broadly. As future work allows, such critiques might be better tailored to specific Internet behaviors, such as the use of sexual media online.

**Table 1.** Comparison of three prominent sets of descriptive criteria for Internet addiction using items from assessment instruments

	Griffiths (2005)	Young (1998b)	Tao et al. (2010)
Salience/Preoccupation	“dominates their thinking (preoccupations and cognitive distortions), feelings (cravings) and behaviour”	- Feel preoccupied with the Internet when off-line or fantasize about being online?	“thinking about previous online activity”
(Negative) Mood management	“use ... behaviours as a way of producing a reliable and consistent shift in their mood state as a coping strategy to ... make themselves feel better”	- Do you block disturbing thoughts about your life with soothing thoughts of the Internet? - Fear that life without the Internet would be boring, empty and joyless?	“uses the internet to escape or relieve a dysphoric mood”
Tolerance	“increasing amounts of the particular activity are required to achieve the former effects”	- Find that you stay online longer than you intended?	“marked increase in internet use required to achieve satisfaction”
Withdrawal	“unpleasant feeling states and/or physical effects which occur when the particular activity is discontinued or suddenly reduced”	- Feel depressed, moody, or nervous when you are offline, which goes away once you are back online?	“manifested by a dysphoric mood, anxiety, irritability and boredom after several days without internet activity”
External consequences/ Conflict	“conflicts between the addict and those around them (interpersonal conflict) or from within the individual themselves (intrapsychic conflict) which are concerned with the particular activity”	- Does your work suffer (e.g., post poning things, not meeting deadlines, etc.) because of the amount of time you spend online? - Does your job performance or pro ductivity suffer because of the Internet? - Choose to spend more time online over going out with others? - Do you prefer excitement of the Internet to intimacy with your part ner? - Neglect household chores to spend more time online? - Lose sleep due to late night log-ins? - Do you check your E-mail before something else that you need to do? - Snap, yell, or act annoyed if some one bothers you while you are online? - Do others in your life complain to you about the amount of time you spend online?	“loss of interests, previous hobbies, entertainment as a direct result of, and with the exception of, internet use” or “deception of actual costs/time of internet involvement to family members, therapist and others” “continued excessive use of internet despite knowledge of having a persistent or recurrent physical or psychological problems likely to have been caused or exacerbated by internet use”
Relapse/Control	“tendency for repeated reversions to earlier patterns of the particular activity to recur”	- Try to cut down the amount of time you spend online and fail? - Find yourself saying “Just a few more minutes” when online?	“persistent desire and/or unsuccessful attempts to control, cut back or discontinue internet use”
Craving/Anticipation		- Do you find yourself anticipating when you go online again?	“anticipation of the next online session” or “a strong desire for the internet”
Lying/Hiding use		- Do you become defensive or secre tive when anyone asks you what you do online? - Try to hide how long you’ve been online?	

## A CRITICAL REVIEW OF POPULAR CRITERIA

Existing critiques of addictive behaviors include IA (Widyanto & Griffiths, 2006), sex addiction (Ley, Prause & Finn, 2014; Moser, 2011), behavioral addictions in general (Potenza, 2006), and impulse control disorders (Dell’Osso, Altamura, Allen, Marazziti & Hollander, 2006). The current critique differs by examining each of the criteria already proposed for IA, rather than attempting to create a new catalog of IA symptoms as many previous publications have proposed new criteria (Chakraborty, Basu & Vijaya Kumar, 2010; Shaffer, Hall & Bilt, 2000; Wood, 2007). Also, here we build on experimental findings to suggest new criteria that would provide stronger evidence of pathology than do the existing proposed criteria. This approach is consistent with the recent recommendation to move away from clustered criteria in a research context (Insel, 2013).

We critique each of the criteria proposed for behavioral addiction individually by the example of IA criteria reviewed above (see Table 1). While the cited authors were not unanimous in referring to the Internet as “addictive”, they are all proposing diagnostic criteria to identify - and thus name - a specific disease. Thus, at a minimum, all the models studied here share the assumption that the Internet can produce a qualitative shift to a diseased state in humans. While clearly a single criterion by itself would be sufficient to identify a disorder, a full analysis of all possible counts and combinations of criteria is beyond the scope of this work.

## NEGATIVE CONSEQUENCES

Most, if not all, models of behavioral addictions seem to agree that negative outcomes are necessary criteria of a disease state. In the proposed category of sexual addiction, this may include the loss of a primary intimate relationship due to infidelity (Schneider, Corley & Irons, 1998), while problem gambling has included time lost from work/school and arguments with cohabitants over gambling behaviors (Lesieur & Blume, 1987). Online video gaming addicts reported more marital difficulties (odds ratio = 4.61) and work difficulties (odds ratio = 4.42), saw fewer friends (odds ratio *—* 5.78), and missed more financial obligations (odds ratio *—* 6.05) than those who game online in a non-addictive manner (Achab et al., 2011).

The negative consequences criterion has strong face validity, as it is difficult to imagine someone voluntarily choosing to suffer negative consequences. Mental illness, such as substance addiction, is often inferred from behavioral tenacity in the face of these negative consequences. However, research on decision-making consistently demonstrates circumstances under which healthy people engage in non-optimal, and often ultimately detrimental, behaviors. For example, the Iowa Gambling task is a card-drawing game in which healthy players have been shown to have a preference for drawing from a deck that loses money (Lin, Chiu, Lee & Hsieh, 2007). Specifically, players without any pathology tend to choose one (of four) card decks (Deck B) that provide a series of small wins, but also large infrequent losses, that result in overall loss. In this example, reframing negative consequences as the result of non-optimal decision-making might well be the more parsimonious approach to interpreting the behavior.

## LOSS OF CONTROL AND/OR RELAPSE

As with negative consequences, a (perceived) lack of control of activities on the Internet is said to accompany Internet addictions. Items assessing controllability typically ask about efforts to reduce use. Surprisingly, such questions accounted for only small (6%) proportions of variance (as opposed to 9% negative effects, and 35% salience) in a survey measure of Internet use problems (Widyanto, Griffiths, Brunsden & McMurran, 2007). Interestingly, a perceived lack of control does not consistently emerge as predictive of problems in self-report assessments used to investigate IA (Widyanto et al., 2011).

Laboratory studies have found more convincingly that those individuals with Internet use problems might have problems with self-regulation. Poor decision making relative to controls has been demonstrated in problematic Internet gamers relative to controls in a dice game (Pawlikowski & Brand, 2011) and in inhibition trials such as go/no-go sequences (Littel et al., 2012). The poor control has been attributed to Internet addicts’ relatively enhanced sensitivity to rewards and their insensitivity to punishments in general decision making tasks (Dong, Huang & Du, 2011). Another way of thinking about controllability is the ability to change one’s own emotional state, now commonly known as affective regulation. For example, a number of studies have highlighted a relationship between decreased regulation abilities and the increased risk of proposed behavioral addictions. In a Turkish study 6% of the variability of Internet use problems was explained by self-reported emotion management skills (Oktan, 2011). Tokunaga and Rains (2010) noted that the ability to regulate affect predicted time using the Internet, whereas depression, loneliness, or social anxiety did not.

Controllability of the addictive behavior in IA and various other addiction models is often mentioned as a key diagnostic factor, possibly because it ascribes fault to a disease and not to the affected individual. However, there is no reason that behaviors cannot be both destructive to the individual and voluntary (for a review, see Heyman, 2009). If an inability to control Internet behaviors could be demonstrated convincingly (beyond self-reports), this would be important and consistent with a disease model. In addition to the perception of feeling out of control, though, it would be important to demonstrate that, even in the presence of valued alternative reinforcers, the addictive Internet behaviors could actually not be stopped. For example, a person might enjoy a strong sex drive, yet decline opportunities to behave sexually with an appropriate, desirable partner in favor of Internet use.

## PREOCCUPATION

In the Internet addiction model, preoccupation generally refers to obsessive and continuous thoughts about Internet activities that contribute to the negative outcomes associated with problem use. Preoccupation is thought to arise as a part of the self-regulation failure accompanying problem Internet use, and is cited as a primary indicator of withdrawal (Caplan, 2010). At least one paper includes time spent in the activity as evidence of preoccupation (Shapira et al., 2003). Time spent in the activity is discussed in other sections in the current review (see below), so will not be discussed further in this context. General measures of the ability to control one’s thoughts do exist in both self-report (Wells & Davies, 1994) and (controversially) laboratory task analogues (Davison, Vogel & Coffman, 1997). However, instruments more specific to Internet problems are generally used with questions such as “When not online, I wonder what is happening online” (Caplan, 2002).

Unlike other addiction criteria, preoccupation with the Internet is a weak indicator of pathology, an intense hobby might just as well lead to preoccupation (Hellman, Schoenmakers, Nordstrom & van Hoist, 2013). Also, the tendency to assess “preoccupation” with novel questionnaires prevents accurate comparisons with other studies. Such comparisons are needed to determine whether thoughts about the Internet exceed thoughts typical of other non-pathological activities, such as hobbies; finally, reliance on self-reported risks over- and under-reported by clients (Beard & Wolf, 2001). While a number of improvements could be made in the assessment of preoccupation, it remains unclear how this would support a disease model of Internet use.

## MOOD MANAGEMENT

In addition to the lack of control discussed in the context of affect self-regulation, engaging in behaviors to manage mood has been characterized as part of the disease model in behavioral addictions. Use of media has been characterized as motivated “to minimize aversion and maximize elation (p. 159)” (Mastro, Eastin & Tamborini, 2002). One author (Zillman, 1988) refers to this as selective exposure, wherein a person selects online media to maintain excitatory homeostasis between over- and under-stimulation. Some support has been claimed for this model by those who are induced to boredom subsequently generating more Internet site hits during browsing (Mastro et al., 2002). In a nationally representative sample, 8.3% of adults reported using the Internet to alleviate negative mood or to escape life’s problems (Aboujaoude, Koran, Gamel, Large & Serpe, 2006).

If online activity helps a person cope effectively with negative affect, it is unclear why such a strategy automatically becomes a criterion for addiction. In fact, the Internet has been equally lauded for introducing new avenues for social support to reduce negative affect (Lamberg, 2003) and to develop social competence (Saunders & Chester, 2008). Furthermore, greater use of the Internet for support has been associated with greater feelings of pride in young women facing cancer (Seçkin, 2011) and providing communication about sexual safety for isolated men who have sex with men (Rhodes, 2004), among other examples. It may be that there are more effective methods of coping with negative affect than engaging in online activities or that some Internet activities are more beneficial than others. Hence, Internet use as mood management has to be considered in context, as the Internet can be used as an effective coping tool.

## TOLERANCE OR WITHDRAWAL

The concepts of tolerance (requiring more of the stimulus to get the same result) and withdrawal (experiencing negative consequences if you stop) have roots in the substance-based approach to addiction. While withdrawal is mentioned infrequently as occurring with cessation of problem gambling (Fisher, 1992), no biological change consistent with reported withdrawal experiences has been demonstrated in gambling or Internet use.

Tolerance is sometimes viewed as neither sufficient nor necessary to support a disease state, even within substance use disorders (Langenbucher et al., 2000). Authors writing about IA sometimes struggle with these two components. Besides Young’s discussed IAT, conceptualizations such as the Compulsive Internet Use Scale (Meerkerk et al., 2009) and ICD-10 Gambling criteria do not include tolerance as a criterion. The validation study by Meerkerk (2007) found little evidence of tolerance, which has resulted in its removal from the CIUS instrument. Although both Tao and Griffiths (and the current phrasing of DSM-5’s Internet gaming disorder) indicate this criterion of tolerance as a component for identification, it remains unclear how ‘marked increase in Internet use to achieve satisfaction’ would manifest physiologically with IA. Relying on patient reports of distress as evidence of withdrawal or tolerance appears to be weak support (Pies, 2009). In short, tolerance and withdrawal are neither (1) the most replicable aspects of substance use problems nor (2) identified clearly in problem Internet users. Thus, tolerance and withdrawal symptoms do not appear to support an addiction model of high Internet use.

## CRAVING OR ANTICIPATION

Craving has been described as the “anticipation of pleasurable relief and a purely subjective phenomenon (Marlatt, 1987, p. 42). Assessments of cravings, then, are almost exclusively based on self-reports, e.g., separating hedonic desire aspects of craving (Caselli, Soliani & Spada, 2012). Also, craving seems to be an experience central to feeling that one is addicted, since craving is often labeled and believed to be a real phenomenon by patients (Kozlowski & Wilkinson, 1987). This is problematic, because it is tautological to suggest that a problem behavior is due to “craving”, and that craving is evidence that the behavior is problematic (Heyman, 2011). Also, research participants’ understanding of the term “craving” has been shown to differ markedly from researchers’ interpretations of the word (Kozlowski & Wilkinson, 1987). Despite this, many researchers have associated craving criteria with behavioral addictions (Armstrong, Phillips & Saling, 2000; Freeman, 2008; Han, Hwang & Renshaw, 2010; Stoeber, Harvey, Ward & Childs, 2011), including IA. For example, those at higher risk for IA have been reported to exhibit lower peripheral temperature and higher respiratory rate when surfing the Internet as compared to resting baseline (Lu, Wang & Huang, 2010). Such physiological indices are non-specific, so they cannot be interpreted as direct evidence of craving.

More recently, fMRI studies have reported that areas of the brain active during drug craving are also active when craving online video games (Ko et al., 2009), and that drug treatment of gaming addicts reduces activation of brain areas associated with craving (Han et al., 2010). Of course, brain areas are not process specific. These areas may simply reflect wanting or liking the substance or activity, or even internal conflict about the substance or activity portrayed. While liking Internet use is necessary early in use to parallel other addictions, it is not sufficient evidence of a diseased state. The reason why liking is not sufficient evidence is that addictive use is characterized by a shift away from liking to craving (Robinson & Berridge, 2000). Moving forward, changes in craving a behavior certainly might predict the increased likelihood of the occurrence of behaviors; however, at present, evidence in this area is limited to the predictive utility of behavioral intentions (Webb & Sheeran, 2006), and changes in craving have not been convincingly linked to changes in behavior. In summary, characterizing craving - or even “high” craving levels, if such an assessment could be made - appears to be insufficient evidence of a disease state.

## TIME CUT-OFFS

Many behaviors could be described as harmful due to excessive involvement (i.e., procrastination, insufficient or excessive exercise, over-eating) without warranting a “disease” label. Behaviors might be usefully defined as problematic by their frequency, but how to quantify “too much” or “too frequent” is often a major point of contention (Weinstein & Lejoyeux, 2010). For example, excesses might be quantified as total orgasm outlet(s) per week for sexual behaviors (Kafka, 1991), hours spent in the gym for exercise (Lejoyeux, Avril, Richoux, Embouazza & Nivoli, 2008), or the amount of specifically forbidden foods consumed for eating problems (Tuomisto et al., 1999). In the case of IA, hours spent online is typically used as one indicator of problematic behaviors (Armstrong et al., 2000). Debate exists as to whether time spent on the activity is a good indicator of having a problem. For example, a nationally representative sample concluded that problem video gaming (online or offline) was not predicted well by the time spent on the activity alone (Gentile, 2009). Moreover, spending time on the Internet is not in and by itself a pathological or even a negative activity. Given this unresolved difficulty of establishing relevance and relevant behavioral cut-offs, time spent alone seems a weak candidate for establishing diseased behavior.

## PROMISING FUTURE DIRECTIONS FOR RESEARCH

Is there a more useful way to think about problem Internet use than an addictions framework? This final section identifies continuum approaches that might prove more fruitful than the categorical approaches used to date (Insel, 2013). Specifically, existing research could be understood within other frameworks that are not addictions. Identifying a model that better fits the existing data would help pinpoint mechanisms supporting the Internet behaviors. Characterizing the nature of Internet behaviors could improve the efficacy of interventions for those who wish to reduce their Internet use.

## COMORBIDITY WITH OTHER PSYCHOPATHOLOGY SUGGESTS UNDERLYING, COMMON VULNERABILITIES

Some researchers try to support the case for IA by demonstrating that IA co-occurs with other Axis-I psychopa-thology (Carli et al., 2013). However, what comorbidity actually implies is a common vulnerability (e.g., impulsivity, cognitive factors), which is made difficult to specify by the use of diagnostic categories. For example, Internet use has been found to vary with levels of depression in a longitudinal study (Kraut et al., 1998), but this may simply reflect the reduction in (real-life) socializing common in several mood disorders. In another example, individuals who are socially anxious use the Internet at higher rates than those who are not socially anxious to reduce the risks of face-to-face interactions (Lee & Stapinski, 2012). Similar points were raised by Wood (2007), who argued that behaviors proposed to be addictive may instead represent failure to cope with an underlying problem, and do not warrant a separate diagnostic category by themselves.

Davis (2001) proposes a cognitive behavioral model of IA focused on what he describes as maladaptive thoughts as the main source of pathological Internet use. These thoughts grow out of an underlying psychopathology, which the authors acknowledge as potentially problematic in ascribing the problem to the Internet use. This psychopathology (i.e. depression, social anxiety, substance dependence), coupled with reinforcing experiences on the Internet, is argued to develop the maladaptive cognitions that maintain overuse and other problematic behaviors. For example, someone might post a comment in an online forum that another may respond positively to, which might lead the original poster to feel socially skilled or intelligent. As variable ratio reinforcement continues with periodic positive reinforcement from other posters, the Internet user is rewarded to check and update the forums frequently. This model has garnered some empirical support (Caplan, 2010). For example, those who were more likely to report using the Internet to improve their mood were more likely also to report failures judging and limiting their Internet use time. Rather than delineating the boundaries of Internet addiction diagnoses, it would be more useful to characterize the individual differences that predispose some people to learn to use the Internet very often. *Summary point (1): Comorbidity with other psychopathology alone does not provide evidence of a unique and separate psychopathology. Focusing on its role as suggesting underlying problems in basic functions (e.g., learning) to be studied further might be more fruitful*.

## BUILDING NEUROIMAGING MODELS, NOT NEUROIMAGING EVIDENCE

Neuroimaging studies have recently begun to emerge in the IA field (Kuss & Griffiths, 2012; Weinstein & Lejoyeux, 2013). One study found that experienced Internet users exhibited more than a twofold greater voxel of activation in the brain in general than did non- or infrequent Internet users (21,782 versus 8,646 total activated voxels) when performing an Internet search task (Small, Moody, Siddarth & Bookheimer, 2009). Specifically, the additional activation appeared in frontal pole, right anterior temporal cortex, the anterior and posterior cingulate, and the right and left hippocampus. Such studies suggest that task repetition over time leads to greater cognitive efficiency, which also is reflected by lower activations following mental training. The length of reported problems with IA has been shown to be inversely proportional to gray matter volume in the dorsolateral prefrontal cortex, rostral anterior cingulate cortex, and supplementary motor area (Yuan et al., 2011). Descriptions of the samples demonstrate alternative interpretations: the first study (Small et al., 2009) described their participants as either Internet “savvy” or “naive”, whereas the second (Yuan et al., 2011) described participants as having “Internet addiction disorder.” The former is an example of building a model of high-frequency Internet use, whereas the latter reified preconceived categories. The savvy/naive distinction reflected the fact that the frequency of use alone can lead to differences. In Yuan et al., a large difference in the “control” and “disorder” groups was the hours of daily Internet use, yet the higher use group was conceptualized as representing a qualitatively distinct problem. Although the authors wish to argue that there is a qualitatively distinct “disorder” group, no data were presented to demonstrate that a pathology, and not merely more frequent use, contributed to the brain differences reported.

Neuroimaging research is, of course, limited by the lack of causal inference and, relatedly, potential third-variable explanations. For example, gray matter volume in these areas also has been related to IQ (van den Bos, Crone & Güroðlu, 2012), in DLPFC to depression vulnerability (Amico et al., 2011), in supplementary motor area in response to motor training (Taubert, Lohmann, Margulies, Villringer & Ragert, 2011), and in rACC associated with suicidality (Wagner et al., 2011). Such variables may better account for the apparent relationship between gray matter and Internet use, and need to be controlled in analyses.

Substances change brain structure and function, which has been linked to reported problems of addiction (Leshner, 1997). Some authors have tried to suggest a similarity with behaviors, which certainly also have the capability of changing brain structure and function; however, this is merely evidence of learning, not addiction (Heyman, 2009). Behavioral addictions research appears poised to commit this error too. For example, Hou et al. (2012) argued that their findings suggest IA was associated with broad dysfunctions in dopaminergic brain systems. Others have reported decreased gray matter volume and greater fractional anisotropy (Yuan et al., 2011; Zhou et al., 2011) as well as abnormal white matter tracts (Lin et al., 2012) in Internet addicts. These non-specific findings may help characterize why some people enjoy the Internet more than others, but this is not sufficient evidence for an independent diagnosis so much as simply a catalog of possible commonalities.

*Summary point (2): Differences in the activity of brain areas are not direct evidence of any pathology, but neuroimaging might be used to model relationships between Internet use and brain function*.

## IDENTIFYING MECHANISMS OF INTERNET USE SHIFTS

Evidence of enhanced reward responsivity has been investigated in relation to IA. Specifically, some people may simply respond more to rewards regardless of whether that reward is sex, drugs, or Internet behaviors. For example, rats who display greater incentive sensitization to food cues are the same rats who become motivated to self-administer cocaine (Saunders & Robinson, 2010). Rats bred to be sensation seeking also display heightened sexual behaviors (Cummings, Clinton, Perry, Akil & Becker, 2013). With respect to IA, some have pointed to a greater prevalence of dopamine polymorphisms in excessive Internet gamers as evidence of reward dependency (Han et al., 2011). Additional studies using PET have shown decreased D2 receptor availability in portions of striatum in excessive gamers compared to controls (Kim et al., 2011), enhanced glucose metabolism in areas related to reward processing (Park, Kim, Bang, Yoon & Cho, 2010), and greater activation of areas indicative of reward processing to game stimuli in excessive Internet gamers (Han et al., 2011). A small study found no difference in BDNF, thought to contribute to craving for drugs following cues, and between IA and controls (Geisel et al., 2013).

These findings are inconsistent with the speculation that those with IA demonstrate tolerance, which would require Internet activities to become less rewarding. Surprisingly, however, these studies have not attempted to link findings to specific behaviors or cognitions, only diagnoses. Thus, it is unclear whether the decreased reward might be occurring due to exposure history, cue type, individual differences in general reward sensitivity, or something else. A more dimensional approach might examine how reward sensitivity to Internet cues versus Internet use changes during different levels of sleep deprivation. This level of specification will help pinpoint what actually contributes to high frequency use and, hence, what might most impact future use. For example, one useful study using bupropion investigated distinguished changes in video game cravings reported and hours of use as continuous variables (Han et al., 2010) will allow integration with cravings and use research in related domains.

Reward sensitivity is non-specific to addiction and may represent a useful dimension to characterize frequent Internet use. For example, personality variables also modulate reward response in the same brain areas (e.g., ventral striatum [Simon et al., 2010]). Goodman (2008) pointed out that increased liking of a behavior is insufficient evidence for its status as an addiction. Another study suggested that such activation was indicative of reward from novelty alone, which may better explain apparent modulations of these brain areas in drug abusers (Bevins, 2001). Pharmacologically increasing dopamine bioavailability also increases expectations of the hedonic value of many different activities that have not been suggested to be addictive (Sharot, Shiner, Brown, Fan & Dolan, 2009), and decreasing dopamine decreases activation of reward areas to chocolate cues (McCabe, Huber, Harmer & Cowen, 2011) in non-pathological people. While enhanced reward processing is not a good indicator of disease, it may be a very useful dimension for characterizing variability in Internet use. For example, some have used reward responsiveness to show a shift in substance use from hedonic liking to more motivated, less hedonic craving (Robinson & Berridge, 2001). Monitoring change in reward responsivity over different levels of Internet use, or attempts to decrease engagement with Internet cues, would identify clearer targets for interventions than predictors of a hypothetical disease state.

*Summary point (3): Shifts in the hedonic experience of Internet cues predictive of use changes might be more useful in changing Internet behaviors than a discrete addiction*.

## AN ALTERNATIVE PATH: AWAY FROM DISEASE, TOWARD BEHAVIORS

Internet behaviors have been described as “problematic”, “excessive”, “addiction”, “dependence”, “pathological”, “impulsive”, “compulsive”, or “abnormal”, or prefixed as “hyper-” to delineate some disease state. Notably absent are behaviorally specific, statistically descriptive terms, such as “high-frequency Internet use”. Additionally, many interventions already exist to change the frequency of specific behaviors, and the frequency and intensity of the behavior is often a main problem in reported cases of addiction.

Taking video games as an example, the amount of time spent playing video games has been associated with obesity (Vandewater, Shim & Caplovitz, 2004). Thus, it might make sense to try to reduce the time spent playing video games in order to prevent snacking that can occur while playing games. Simple behavioral, token economy interventions have proven successful for reducing the hours of television viewed (Schmidt et al., 2012) and could be adapted to modify gaming and other Internet involvement. Some might ob-ject that such a focus on behavior fails to treat some underlying cause of the behavior, which could result in relapse. A more straightforward focus on behavior and dimensions underlying behavior change, rather than development of another addiction model, appears reasonable.

Furthermore, attributing Internet behaviors to a disease state can be harmful. A diagnosis can be comforting, providing a label to describe isolating experiences and validating the challenges involved in changing a behavior (Rubin, 2000). However, diagnoses also can make change more difficult. For example, the primary mechanism of change when using biofeedback to treat chronic pain is increased self-efficacy, not changes in actual muscle tension (Holroyd, 2002). Addiction model treatments that teach the patient they are not in control of their addiction might actually reduce their self-efficacy and make behavior change less likely. A pragmatic focus on behavioral modification has already generated positive feedback from psychologists treating a small group of self-diagnosed IA patients (van Rooij, Zinn, Schoenmakers & Mheen, 2012).

This review did not address some potentially important aspects of Internet behaviors in a desire to limit the scope of the review. For example, Internet use can be characterized as varying on social involvement (e.g., Facebook versus solitaire), immediate financial risk (e.g., online poker with bitcoin versus blogs), or social acceptability (e.g., perusing sexual videos versus nature photography). It may be that specific Internet behaviors follow addictive patterns, whereas Internet behaviors in general do not. Also, the three models reviewed were an editorial choice to provide further insight into a few of the more popular models. An alternative approach would have been to conduct a systematic review of publications about Internet behaviors to quantify, in greater breadth, the extent to which the different symptoms actually are used.

In summary, many challenges exist to conceptualizing problem Internet behaviors as a disease. While progress is being made setting up large-scale, longitudinal studies, such as a recent Europe-wide (eleven countries, *N* = 11,956) investigation (Durkee et al., 2012), the otherwise impressive study continues to rely on an eight-item version of the Young Internet Addiction questionnaire. The failure to develop and use strongly theoretical measures limits the strength of these investigations. This questionnaire continues to float on face validity to classify users into a “pathological” group using an arbitrary cut-off score. We believe that this leaves ample room for improvement in the field. It also provides researchers with an opportunity for methodological improvement in the field by focusing on theoretical modeling, experimental results, and a dimensional approach.
